# Fetal *ERAP2 *variation is associated with preeclampsia in African Americans in a case-control study

**DOI:** 10.1186/1471-2350-12-64

**Published:** 2011-05-11

**Authors:** Lori D Hill, DaShaunda D Hilliard, Timothy P York, Sindhu Srinivas, Juan P Kusanovic, Ricardo Gomez, Michal A Elovitz, Roberto Romero, Jerome F Strauss

**Affiliations:** 1 Department of Obstetrics and Gynecology and Center on Health Disparities, Virginia Commonwealth University School of Medicine, Richmond, VA, 23298, United States; 2 Department of Human and Molecular Genetics, Virginia Commonwealth University School of Medicine, Richmond, VA, 23298, United States; 3 Maternal and Child Health Research Program, Department of Obstetrics & Gynecology, University of Pennsylvania School of Medicine, Philadelphia, PA, 10104, United States; 4 Perinatology Research Branch, NICHD/NIH/DHHS, Bethesda and Detroit, MD and MI, 48201, United States; 5 Department of Obstetrics and Gynecology, Sótero del Río Hospital, Santiago, Chile; 6 Department of Obstetrics and Gynecology, Pontifica Universidad Católica de Chile, Santiago, Chile

## Abstract

**Background:**

Preeclampsia affects 3-8% of pregnancies and is a major cause of maternal and perinatal morbidity and mortality worldwide. This complex disorder is characterized by alterations in the immune and vascular systems and involves multiple organs. There is strong evidence for a genetic contribution to preeclampsia. Two different single nucleotide polymorphisms (SNPs) in the *endoplasmic reticulum aminopeptidase 2 (ERAP2) *gene were recently reported to be associated with increased risk for preeclampsia in two different populations. *ERAP2 *is expressed in placental tissue and it is involved in immune responses, inflammation, and blood pressure regulation; making it is an attractive preeclampsia candidate gene. Furthermore, *ERAP2 *expression is altered in first trimester placentas of women destined to develop preeclampsia.

**Methods:**

A case-control design was used to test for associations between two SNPs in *ERAP2*, rs2549782 and rs17408150, and preeclampsia status in 1103 Chilean maternal-fetal dyads and 1637 unpaired African American samples (836 maternal, 837 fetal).

**Results:**

We found that the fetal minor allele (G) of rs2549782 was associated with an increased risk for preeclampsia in the African American population (*P *= 0.009), but not in the Chilean population. We found no association between rs17408150 and risk for preeclampsia in the Chilean population. Association between rs17408150 and risk for preeclampsia was not tested in the African American population due to the absence of the minor allele in this population.

**Conclusions:**

We report an association between fetal *ERAP2 *and preeclampsia in an African American population. In conjunction with previous studies, which have found maternal associations with this gene in an Australian/New Zealand population and a Norwegian population, *ERAP2 *has now been associated with preeclampsia in three populations. This provides strong evidence that *ERAP2 *plays a role in the development of preeclampsia.

## Background

Preeclampsia (PE) affects 3-8% of pregnancies worldwide, with rates varying by ethnicity, and leads to potentially devastating complications for both the mother and fetus[1,2]. Preeclampsia is clinically characterized by high blood pressure and proteinuria, usually occurring after 20 weeks of gestation. Although this serious disorder is common during pregnancy, its etiology remains poorly understood[1]. Preeclampsia is considered a disease of the placenta, with shallow trophoblast invasion[3-5] and poor spiral artery remodeling[6-8] being central features of this disorder. It is postulated that immune, vascular, and inflammatory disturbances participate in the placental dysfunction that ultimately produces the preeclampsia phenotype[9].

A genetic susceptibility to preeclampsia has been established with both maternal and fetal genes contributing to disease[2,10-17]. Preeclampsia is a multi-factorial trait, with multiple genes, as well as environmental and social factors contributing to disease risk[18-20]. Johnson *et al*. recently reported that *Endoplasmic reticulum aminopepetidase 2 *(*ERAP2*) was associated with preeclampsia in an Australian/New Zealand family-based study and a Norwegian case-control study of maternal samples[21]. Although *ERAP2 *was associated with risk for preeclampsia in both populations, different polymorphisms of the gene were identified in each group. *ERAP2 *is expressed in the syncytiotrophoblast and it is a member of the oxytocinase subfamily of M1 aminopeptidases, which are known to play a critical role in the maintenance of normal pregnancy[22-24]. Additionally, ERAP2 is involved in the regulation of blood pressure, immune responses, and pro-inflammatory cytokine production[22,25-28]. It was recently shown that *ERAP2 *expression was altered in first trimester placentas of pregnancies destined to develop preeclampsia[29]. The involvement of *ERAP2 *in multiple pathways known to influence the risk for preeclampsia, its expression in placental tissue, and the previously described altered expression of *ERAP2 *in placentas before maternal symptoms developed[29]; suggest that the fetal *ERAP2 *gene contributes to the development of preeclampsia.

In the present study, we investigated whether the previously described associations between *ERAP2 *and risk for preeclampsia [21] replicated in other ethnic groups and extended our study design past maternal only samples to also include fetal samples. We examined the association between *ERAP2 *and risk for preeclampsia in two distinct case-control cohorts: Chilean (1103 maternal-fetal dyads) and African American (836 maternal and 837 fetal samples). We genotyped the two SNPs in *ERAP2*, rs17408150 and rs2549782, that were previously identified as being associated with preeclampsia. Our results demonstrate that the rs2549782 SNP of the fetal *ERAP2 *gene is significantly associated with risk for preeclampsia in the African American population; further suggesting that this gene plays a key role in the development of disease and may provide insight into the disparity between preeclampsia rates between ethnic groups.

## Methods

### Chilean study design and population

A case-control study was initiated by searching the clinical database and bank of biological samples of the Perinatology Research Branch (*Eunice Kennedy Shriver *National Institute of Child Health and Human Development, NIH, DHHS) and included Hispanic women and their neonates in the following groups: 1) Cases - women with preeclampsia and their neonates (*n *= 528 dyads); and 2) Controls - women who delivered at term with a normal pregnancy outcome and their neonates (*n *= 575 dyads). Participants received obstetrical care at the Sótero del Río Hospital in Santiago, Chile (an affiliate of the Pontificia Católica de Chile in Santiago, Chile). Exclusion criteria included: (1) known major fetal anomaly or demise; (2) multi-fetal pregnancy; (3) serious maternal medical illness (renal insufficiency, congestive heart disease, etc.); (4) refusal to provide written informed consent; and (5) a clinical emergency, which prevented counseling of the patient about participating in the study, such as fetal distress or maternal hemorrhage. All women provided written informed consent before collection of the samples. The use of clinical data and collection and utilization of maternal and neonatal blood for research purposes was approved by the Institutional Review Boards of the Sótero del Río Hospital, the *Eunice Kennedy Shriver *National Institute of Child Health and Human Development, NIH, DHHS and Virginia Commonwealth University. Ethnically, the Chilean population is estimated at nearly 95% white and mestizo (mixed white and Amerindian); 3% Amerindian; and 2% other. Mixtures between the conquering Spaniards, largely Andalusians and Basques, and the Mapuches (Araucanians) produced the principle Chilean racial type (2002 census).

### African American study design and population

A case-control study was initiated by searching clinical databases and bank of biological samples at the University of Pennsylvania and the Perinatology Research Branch (*Eunice Kennedy Shriver *National Institute of Child Health and Human Development, NIH, DHHS), at Wayne State University. Study subjects included African American women and neonates in the following groups: 1) Cases - women with preeclampsia (*n *= 424) and neonates born to women with preeclampsia (*n *= 375); and 2) Controls - women who delivered at term with a normal pregnancy outcome (*n *= 412) and neonates delivered at term to women with a normal pregnancy outcome (*n *= 462). Participants in this study received obstetrical care at the University of Pennsylvania Medical Center, Philadelphia, PA or the Hutzel Women's Hospital, Detroit, MI. The criteria for cases, controls, and exclusion of subjects in the African American study were the same as described for the Chilean study. Of the maternal and neonatal subjects identified, 78% of samples were identified as maternal-neonatal dyads. To obtain adequate sample sizes for this study, therefore, maternal and neonatal samples were tested independently and un-paired samples were included in each group. The use of clinical data and collection and utilization of maternal blood, cord blood, and neonatal cheek swabs for research purposes was approved by the Institutional Review Boards of the University of Pennsylvania, Wayne State University, the *Eunice Kennedy Shriver *National Institute of Child Health and Human Development, NIH, DHHS, and Virginia Commonwealth University. African American ethnicity was self-reported for all samples.

### Clinical definitions

Preeclampsia was defined based on the presence of gestational hypertension (systolic blood pressure ≥140 mmHg and/or diastolic blood pressure ≥90 mmHg) and proteinuria (≥300 mg in a 24-hour urine collection, two or more dipstick measurement of 1+, or one or more dipstick measurement ≥2+) according to ACOG[1] and the National High Blood Pressure Education Program[30]. Patients were considered to have a normal pregnancy outcome if they did not have any medical, obstetrical, or surgical complication, and delivered a term neonate (≥37 weeks) of appropriate birth weight for gestational age[31,32] without complications.

### Sample collection

Maternal blood samples were obtained from the mother at the time of enrollment in the protocol. Umbilical cord blood samples or neonate cheek swabs were obtained immediately after delivery. Blood samples were collected with a vacutainer into tubes containing EDTA. The plasma tubes were balanced and centrifuged at 1300*g *for 10 minutes at 4°C to separate cellular components from clear plasma, and the samples were stored at -70°C until assay.

### DNA extraction

DNA was extracted from maternal and cord blood with a Qiagen Autopure system using standard procedures (Qiagen). DNA was extracted from neonate check swabs using traditional methods as previously described[33].

### Genotyping

Single-nucleotide polymorphism analysis was performed using real-time allelic discrimination TaqMan assays (Applied Biosystems) with modifications. All PCR reactions contained 25-75 ng of DNA, 6.25 ul TaqMan Universal Master Mix (Applied Biosystems) (2×), 0.3 ul TaqMan Genotyping Assay (Applied Biosystems) (20×), and water for a final volume of 12.5 ul. Real-time PCR was performed on an ABI 7500 Fast Real-Time PCR Machine (Applied Biosystems) under the following conditions: 50°C for 2 min, 95°C for 10 min, and 40 cycles of amplification (92°C for 15 sec and 60°C for 1 min). For each cycle, the software determined the fluorescent signal from the VIC- or FAM- labeled probe (Applied Biosystems). Allelic discrimination for *ERAP2 *was performed using TaqMan Genotyping assays C___3282749_20 for SNP rs2549782 and C___25649505_10 for SNP rs17408150 (Applied Biosystems).

### Statistical Analysis

Logistic regression in R was used to test for differences in clinical characteristics between disease classes for non-genetic variables. Fisher's exact tests implemented in the PLINK software[34] were used to test individual SNPs for genetic associations with case-control status and to confirm Hardy-Weinberg equilibrium. SNPs with an independent effect were further investigated by multiple logistic regression in R to condition by covariates found to be significantly different between cases and controls in the clinical characteristics analysis. An additive term for the significant SNP(s) was coded as 0, 1, or 2, based on copy number of the minor allele. Allele frequencies from the control groups were used to determine the odds ratios at which our study design had 80% power at an alpha of 0.05. Power calculations were made using the Genetic Power Calculator[35], assuming a 5% disease prevalence.

## Results

### Clinical Characteristics of the Study Populations

Table 1 displays the demographic and clinical characteristics of mothers and neonates from pregnancies with preeclampsia as well as controls. For Chilean subjects, no significant differences were observed in maternal age or fetal sex between groups. Consistent with previous epidemiologic studies, Chilean patients with preeclampsia showed a significantly higher body mass index (BMI) (*P *<0.001) and fewer previous live births (*P *= 0.007). In accordance with preeclampsia resulting in intrauterine growth restriction and indicated preterm birth, offspring born to Chilean women with preeclampsia showed a significantly lower gestational age at delivery and birth weight (*P *< 0.001). Similar results were observed in African American subjects. Maternal age was not significantly different between cases and controls for either the maternal or the fetal study groups, whereas, gestational age at delivery and birth weight were significantly different between cases and controls for both groups (*P *< 0.001). Additionally, in the fetal group, mothers with preeclampsia showed a significantly higher BMI (*P *= 0.049) and fewer previous live births (*P *= 0.040). Although these measures were not significant in the maternal study group, they were trending in the same direction. In the fetal group, there were significantly more female neonates than male (*P *= 0.024). Significant differences in fetal sex have been reported in the literature, but results vary with some studies reporting a bias towards male fetuses, some reporting a bias towards female fetuses, and still others reporting no differences in fetal sex in association with preeclampsia[36-44]. No significant difference in fetal sex was observed between cases and controls in the maternal study group.

**Table 1:** Maternal and fetal characteristics of pregnancies diagnosed with preeclampsia and controls.

Population		Preeclampsia	Controls	*P*-value
Chilean	Number of dyads	528	575	-
Maternal-Fetal Dyads	Maternal Age (*years)*	26.3 (7.5)	26.1 (6.2)	0.692
	BMI (*kg/m^2^)*	26.4 (5.4)	24.5 (4.4)	< 0.001
	Previous live births	0.80 (1.19)	0.99 (1.08)	0.007
	Birthweight (*grams)*	2805.7 (815.7)	3423.2 (303.0)	< 0.001
	Gestational age at delivery (*weeks)*	36.8 (3.4)	39.7 (1.1)	< 0.001
	Fetal sex (*% female)*	45.8	53.3	0.492

African American	Number of subjects	424	412	-
Maternal	Maternal Age (*years)*	26.0 (6.3)	25.3 (5.9)	0.100
	BMI (*kg/m^2^)*	30.9 (8.7)	29.7 (7.9)	0.070
	Previous live births	3.2 (2.3)	3.3 (2.0)	0.529
	Birthweight (*grams)*	2431.1 (893.8)	3292.1 (462.4)	< 0.001
	Gestational age at delivery (*weeks)*	36.0 (3.7)	39.5 (1.3)	< 0.001
	Fetal sex (*% female)*	52.4	48.4	0.253

African American	Number of subjects	375	462	-
Fetal	Maternal Age (*years)*	25.8 (6.5)	25.8 (6.1)	0.947
	BMI (*kg/m^2^)*	31.0 (8.5)	29.8 (7.9)	0.049
	Previous live births	3.1 (2.2)	3.4 (2.1)	0.040
	Birthweight (*grams)*	2490.3 (851.8)	3294.7 (469.7)	< 0.001
	Gestational age at delivery (*weeks)*	36.2 (3.4)	39.5 (1.2)	< 0.001
	Fetal sex (*% female)*	54.8	47.0	0.024

Data are presented as means (SD). BMI, body mass index.

### Chilean Population

The minor allele (G) frequencies for rs2549782 in maternal and fetal samples were 0.3386 and 0.3292, respectively. The minor "A" allele frequencies for rs17408150 in maternal and fetal samples were 0.0422 and 0.0395 respectively. The minor allele frequencies are consistent with published data and the Johnson *et al*. study[21,45]. Single SNP analysis revealed no associations between *ERAP2 *polymorphisms rs2549782 and rs17408150 and preeclampsia in either maternal or fetal samples (Table 2). All SNPs were found to be in Hardy-Weinberg equilibrium in the maternal and fetal control samples and no substantial linkage disequilibrium was observed (R^2 ^= 0.087 and 0.072, respectively).

**Table 2:** *ERAP2 *Allelic analysis for maternal and fetal samples with and without preeclampsia.

Population	*ERAP2 *SNP	Genotype (count)	Minor Allele	Frequency Preeclampsia	Frequency Controls	*P*-value	Odds Ratio (95% C.I.)
Chilean	Maternal						
	rs2549782	GG (135)	G	0.330	0.347	0.393	0.925 (0.775, 1.104)
		TG (477)					
		TT (491)					
	rs17408150	AA (2)	A	0.044	0.041	0.752	1.069 (0.706, 1.619)
		TA (89)					
		TT (1012)					
	Fetal						
	rs2549782	GG (124)	G	0.333	0.326	0.751	1.033 (0.865, 1.234)
		TG (477)					
		TT (500)					
	rs17408150	AA (0)	A	0.040	0.039	1.000	1.021 (0.665, 1.568)
		TA (87)					
		TT (1014)					
African	Maternal						
American	rs2549782	GG (147)	G	0.429	0.391	0.133	1.166 (0.958, 1.420)
		TG (383)					
		TT (295)					
	Fetal						
	rs2549782	GG (114)	G	0.435	0.369	0.009	1.320 (1.075, 1.619)
		TG (387)					
		TT (268)					

SNP, single nucleotide polymorphism; C.I., confidence interval. The minor allele (G) of rs2549782 was found significantly more frequently in cases than controls in African American fetal samples.

### African American Population

The minor allele (G) frequencies for rs2549782 in maternal and fetal samples were 0.4103 and 0.3990 respectively. The minor allele frequencies are consistent with published data and the Johnson *et al*. study[21,45]. We did not genotype rs17408150 in this population because the minor "A" allele is reported to be < 1.0% in individuals of African descent[45].

To establish the genetic similarity between the University of Pennsylvania Medical Center and Hutzel Women's Hospital African American samples, and determine if these groups were appropriately combined into a single study population, we compared allele frequencies for three genes: *ERAP2, MTHFR*, and *COMT*. Allele frequencies of both *COMT *and *MTHFR *are not only known to differ among major ethnic categories, but substantial variation has also been demonstrated in subpopulations of each, including African American[45-51]. Genotypes for *MTHFR *and *COMT *were readily available for our samples and based on their aforementioned ethnic variation, they represented ideal genes for the genetic comparison of the two African American sample collection locations. Minor allele frequencies for *ERAP2*, *MTHFR*, and *COMT *were comparable between both African American study sites (Table 3). Additionally, the same *COMT *haplotype structure was identified in each group and the haplotype frequencies were comparable. The genetic similarity of the two groups across six variable SNPs and COMT haplotype structure and frequency, supported combing the groups into a single African American study population.

**Table 3:** Genotype and Haplotype frequencies for *ERAP2, MTHFR*, and *COMT *for African American samples.

				Minor Allele Frequency	
				
Group	Gene	SNP/Haplotype	Minor Allele	Pennsylvania	Michigan
Maternal	*ERAP2*	rs2549782	G	0.397	0.374
	*MTHFR*	rs1801133	T	0.112	0.102
	*COMT*	rs6269	G	0.393	0.384
		rs4633	T	0.292	0.316
		rs4818	G	0.200	0.215
		rs4680	A	0.276	0.291
		ATCA		0.249	0.270
		GCCA		0.027	0.021
		GCGG		0.163	0.169
		ACGG		0.037	0.048
		ATCG		0.043	0.045
		GCCG		0.203	0.194
		ACCG		0.278	0.253
Fetal	*ERAP2*	rs2549782	G	0.359	0.424
	*MTHFR*	rs1801133	T	0.120	0.133
	*COMT*	rs6269	G	0.408	0.425
		rs4633	T	0.319	0.300
		rs4818	G	0.214	0.167
		rs4680	A	0.292	0.308
		ATCA		0.267	0.257
		GCCA		0.022	0.051
		GCGG		0.175	0.139
		ACGG		0.040	0.028
		ATCG		0.049	0.043
		GCCG		0.207	0.235
		ACCG		0.240	0.247

African American samples originated from two locations: the University of Pennsylvania Medical Center, PA and Hutzel Women's Hospital, MI. Minor allele frequencies and haplotype frequencies were calculated from control samples only at each location. When comparing locations, no test achieved a significant difference at the 5% level using a Z-test for differences in two independent proportions. SNP, single nucleotide polymorphism. *COMT *single SNP frequencies are listed first, followed by *COMT *haplotypes formed by those SNPs. *COMT *haplotype SNP order: rs6269, rs4633, rs4818, rs4680.

Single SNP analysis yielded a significant association between the fetal rs2549782 and preeclampsia in the African American population (*P *= 0.009), while no association was observed in the maternal SNP (Table 2). Additional multiple logistic regression analysis was performed on the fetal group to adjust for risk factors of preeclampsia (BMI, previous live births, and gravidity) that were found to be significant in the clinical measures analysis (Table 4). rs2549782 remained significant (*P *= 0.012) and was associated with an increased risk for preeclampsia (OR = 1.529; CI: 1.099, 2.128). Of the previously identified clinical measures tested, only the number of previous live births remained significant, with a larger number of previous live births decreasing the risk for preeclampsia (OR = 0.845; CI: 0.744, 0.960). All SNPs were found to be in Hardy-Weinberg equilibrium in the maternal and fetal groups. Finally, we used two methods to confirm that the positive association we observed was not attributed to population stratification based on the different African American sample collection locations. First, multiple logistic regression analysis was performed in R to test whether there was an interaction between the fetal genotype and the sample collection location. No significant association was observed between a location × fetal rs2549782 interaction and the risk for preeclampsia (*P *= 0.098). Second, we performed a cluster analysis in PLINK using a Cochran-Mantel-Haenszel model that tested for overall disease/gene association, while controlling for clusters. After controlling for the sample collection location, the fetal rs2549782 was still significantly associated with an increased risk for preeclampsia (*P *= 0.027; OR = 1.302; CI: 1.029, 1.648). These results, in addition to the absence of evidence for differences in the rates of preeclampsia between African American groups in the United States, justifies combining these samples in this study.

**Table 4:** Logistic regression model for preeclampsia, including presence of the rs2549782 minor allele in African American fetuses.

Term	Estimate (S.E.)	*P*-value	Odds Ratio (95% C.I.)
Fetal rs2549782	0.425 (0.169)	0.012	1.529 (1.099, 2.128)
Maternal BMI	0.021 (0.014)	0.140	1.021 (0.993, 1.049)
Previous live births	- 0.168 (0.065)	0.010	0.845 (0.744, 0.960)
Fetal Sex (% female)	0.355 (0.228)	0.120	1.426 (0.912, 2.231)
Intercept	-1.810 (0.492)	< 0.001	-

S.E., standard error; C.I., confidence interval; BMI, body mass index. The minor allele (G) of rs2549782 significantly increases the risk for preeclampsia in African American fetal samples; after correcting for risk factors identified to modulate risk in this population.

## Discussion

Preeclampsia is one of the leading causes of maternal and perinatal morbidity and mortality worldwide; yet its etiology is poorly understood[1]. It is thought that poor placentation and inadequate maternal blood supply lead to placental hypoxia and the placental release of factors that contribute to intravascular inflammation[52-54], generalized endothelial dysfunction[55-59] and the maternal symptoms. A genetic susceptibility to preeclampsia is well established and genes involved with the immune system, inflammation, hemodynamics, endothelial dysfunction, oxidative stress, and angiogenesis have been associated with preeclampsia[10,15-17]. The identification of genes involved in a variety of physiologic processes reflects the complex nature of this disorder.

It was recently reported by Johnson *et al*. that the *ERAP2 *gene was associated with preeclampsia[21]. They found an association with the rs2549782 SNP in an Australian/New Zealand maternal cohort and the rs17408150 SNP in a Norwegian maternal cohort. In the present study, we sought to test whether there were associations between the two previously identified SNPs in *ERAP2 *and risk for preeclampsia in two distinct ethnic sample sets, Chilean and African American. In contrast to the previous study, we also included fetal samples to determine if the fetal *ERAP2 *gene was associated with risk for preeclampsia. We were motivated to use this design by the fact that placental tissue is of fetal origin and by interest in determining if any genetic association might be attributed to the sharing of alleles between mother and fetus of one-half, in accordance with Mendelian segregation patterns. We found that, in African Americans, the presence of the minor allele (G) of the rs2549782 SNP in the fetal *ERAP2 *gene increased the risk for preeclampsia. We found no associations between the two SNPs in the Chilean population, or the rs2549782 SNP of the maternal *ERAP2 *gene in the African American population.

Preeclampsia is usually diagnosed after 20 weeks of gestation, but it is thought that problems arising early in pregnancy, especially during placentation, are the origin of this disorder. *ERAP2 *is expressed in the syncytiotrophoblast and it has been reported that expression of this gene was down-regulated in first trimester placentas of women who subsequently developed preeclampsia[23,29]. The identification of aberrant gene expression, before maternal symptoms develop, suggests a role for *ERAP2 *early in the disease course.

*ERAP2 *has the potential to contribute to the development of preeclampsia in multiple ways due to its involvement in the regulation of immune responses, pro-inflammatory cytokine production, and blood pressure[22,25-28]. Preeclampsia is associated with a predominant T Helper Cell Type 1 (Th1) immune response, which correlates to poor placentation, inflammation, and endothelial dysfunction[60]. One of the primary roles of ERAP2 is Human Leukocyte Antigen (HLA) trimming of class 1-binding peptides. Decreased levels of HLA-G have been reported in the circulation of women with preeclampsia and reduced cell-surface expression has been reported in trophoblasts[22,27,61,62]. Interferon-gamma (IFN γ) regulates both *ERAP2 *and *ERAP1 *genes and they have been implicated in immune activation and inflammation[28]. ERAP1, which is closely related to and forms complexes with ERAP2[61], also cleaves the cell surface receptors for pro-inflammatory cytokines.

Pregnancy is a pro-inflammatory state, and inflammation is a key regulator of placentation[52,54,63,64]. Although normal pregnancy is pro-inflammatory, preeclampsia is associated with an exaggerated state of systemic inflammation, and aberrant production of placental cytokines has been widely reported [65]. The placental release of pro-inflammatory cytokines, or the pre-existence of increased inflammation in the maternal vasculature, could both contribute to the development of preeclampsia. In addition to being pro-inflammatory, many cytokines also regulate other processes that are important to the establishment and maintenance of pregnancy. Placentation is tightly regulated by the oxygen balance to ensure adequate remodeling of the maternal spiral arteries and sufficient perfusion of the placenta[66]. Hypoxia Inducible Factor 1α (HIF-1α) is a transcription factor that mediates cellular responses to hypoxia and its expression is altered in preeclampsia[67-69]. HIF-1α is regulated through oxygen dependent and independent mechanisms, and several of the cytokines that are modulated by ERAP2 have been shown to participate in the oxygen independent regulation mechanisms[70].

Finally, ERAP2 regulates blood pressure through the renin-angiotensin (RAS) pathway. Specifically, ERAP2 cleaves Angiotensin III and kallidin, both of which are involved in regulating the dilation and constriction of blood vessels[27]. Abnormalities in the processing of these vasoactive substances could be a cause of maternal high blood pressure, but they also might participate in placental hypoxia, which is a key feature of preeclampsia. Defects in the RAS system have been demonstrated both in the maternal system and fetal tissue[71,72], further emphasizing the potential for *ERAP2 *to be involved in the pathophysiology of preeclampsia.

Compared to white women (defined as not African American, Asian, Hispanic, or Native American), Caughey *et al*. found higher rates of preeclampsia among African American women and lower rates among Hispanic women [2]. Additionally, maternal-paternal ethnic discordance was reported to be associated with an increased incidence[2]. This supports the hypothesis that the genetic basis for preeclampsia is heterogeneic. Our results, in conjunction with the findings of Johnson *et al*., provide a potential explanation for the observed differences between ethnic groups[21]. Four ethnic populations were examined between the two studies. Allelic variation between European groups, especially Mediterranean, central Europe, and Scandinavia are well characterized and support that they are distinct populations[45-51]. The Chilean population is representative of a Mediterranean ethnic background, specifically from Spanish decent. *ERAP2 *appears to contribute to the risk for preeclampsia in three of the ethnic groups, with two different allelic variants being associated with risk. Maternal variants increase the risk for preeclampsia in an Australian/New Zealand cohort and a Norwegian cohort[21]. Although preeclampsia is thought to be a placental disorder, the maternal phenotype and, in particular, the susceptibility of the maternal system to disease plays an important role in this disorder[18]. Chronic hypertension, obesity, diabetes, and renal disease, all put a woman at increased risk of developing this disorder. A fetal variant increases the risk for preeclampsia in the African American cohort. Importantly, the placenta is fetal tissue and our results strengthen the argument that primary defects in the placenta play a central role in the development of preeclampsia. Moreover, this finding is consistent with the observation of altered *ERAP2 *expression in placentas from women who developed preeclampsia.

A strength of our study is the inclusion of both maternal and fetal genes, which gives us the ability to discriminate between maternal and fetal genetic effects. The mother and fetus share fifty percent genetic identity so failure to include both maternal and fetal genes in a study creates the potential for a true association with the unmeasured gene to manifest as an observed association with the measured gene based on the correlation between maternal and fetal genotypes. There is also the potential for both the maternal and fetal *ERAP2 *genes to contribute to the risk for preeclampsia in a single ethnic population. By measuring only the maternal genes, an additional fetal association could be missed. Thus, the question still remains whether both maternal and fetal *ERAP2 *contribute to preeclampsia in different ethnic populations where only maternal genes were tested.

A second potential source of variation between ethnicities is the finding that two different SNPs in the *ERAP2 *gene are associated with risk for preeclampsia. Both of these SNPs are missense mutations that are predicted to alter the three-dimensional structure of the protein and damage function. Additionally, rs2549782 resides within the highly conserved zinc-binding domain. While both SNPs are expected reduce enzyme function, they likely alter function to different degrees and are not equivalent mutations. Moreover, the SNPs reside in different domains of the protein and since ERAP2 has multiple functions, the mutations could have significantly different physiologic consequences.

Alternatively, the observed variation could be explained by differences in linkage disequilibrium (LD) structure between populations or failure to account for larger haplotype structure. Although, the SNPs tested in these studies are predicted to alter enzyme function, they might not represent the causal variant in preeclampsia. These populations might share the same causal variant, but that variant could be in LD with different SNPs in each population. Finally, two haplotypes of *ERAP2 *have recently been described that lead to changes in mRNA decay and ultimately Major Histocompatibility Complex (MHC) class I presentation on cell surfaces[73]. The haplotypes are composed of numerous SNPs, with rs2549782 representing one of the four coding SNPs that are considered diagnostic[73]. The frequency of each haplotype was estimated to be 0.5 across multiple ethnic groups and similar patterns of long-range LD were also observed; indicating a single ancestral division of functional significance[73]. Neither our study, nor Johnson et al. included the depth of sequencing necessary to characterize the reported haplotypes.

Our findings did not support a genetic association between *ERAP2 *and the risk for preeclampsia in either the Chilean population or the maternal African American population. However, it should be noted that the present study had limited statistical power to detect very small effects. In the Chilean population, our study was adequately powered to detect Odds Ratios of at least 2.3 for rs17408150 and 1.5-1.7 for rs2549782. In the African American population, our study was adequately powered to detect Odds Ratios of 1.6 - 1.9 for rs2549782. The effect sizes for a single risk factor in a complex disorder are expected to be relatively modest. Furthermore, we only tested for associations between two SNPs in the *ERAP2 *gene so we are unable to rule out the possibility that different variants of this gene are associated with risk for preeclampsia in these populations. Future studies, increasing the number of markers to saturate the maternal and fetal *ERAP2 *genes, are needed to characterize the haplotype structures of each group in order to distinguish between maternal and fetal effects of this gene.

## Conclusions

Our results show that fetal carriage of the minor allele (G) of rs2549782 in the *ERAP2 *gene increases the risk for preeclampsia in African Americans. We found no associations between the maternal rs2549782 SNP of the *ERAP2 *gene and risk for preeclampsia in either the African American or Chilean populations or the rs17408150 SNP of the *ERAP2 *gene and risk for preeclampsia in the Chilean population. The association of rs2549782 with risk for preeclampsia is consistent with findings of a previous study that found an association of maternal *ERAP2 *alleles in an Australian/New Zealand population[21]. The results of our study, in combination with those of Johnson *et al*.[21], describe replicated associations between *ERAP2 *and preeclampsia in three distinct populations. These observations represent an important step in understanding the pathophysiology of preeclampsia and how genetic variation might play a significant role in ethnic differences.

## Competing interests

The authors declare that they have no competing interests.

## Authors' contributions

LDH participated in the design of the study, performed experiments, performed the statistical analysis, and drafted the manuscript. DDH performed experiments. TPY participated in the design of the study, participated in the statistical analysis, and helped to draft the manuscript. JPK provided Chilean and African American purified DNA for use in this study and helped to draft the manuscript. RG coordinated sample collection of Chilean subjects with JPK and RR and helped to draft the manuscript. MAE provided African American DNA for use in this study and helped to draft the manuscript. RR provided Chilean and African American DNA for use in this study and helped to draft the manuscript. JFS conceived of the study, participated in its design and coordinated and helped to draft the manuscript. SS provided African American purified DNA for use in this study and helped to draft the manuscript. All authors have read and approved the final manuscript.

## Pre-publication history

The pre-publication history for this paper can be accessed here:

http://www.biomedcentral.com/1471-2350/12/64/prepub
